# Roadmap for Sex-Responsive Influenza and COVID-19 Vaccine Research in Older Adults

**DOI:** 10.3389/fragi.2022.836642

**Published:** 2022-02-11

**Authors:** Janna R. Shapiro, Rosemary Morgan, Sean X. Leng, Sabra L. Klein

**Affiliations:** ^1^ Department of International Health, Johns Hopkins Bloomberg School of Public Health, Baltimore, MD, United States; ^2^ Division of Geriatric Medicine and Gerontology, Department of Medicine, Johns Hopkins University School of Medicine, Baltimore, MD, United States; ^3^ W. Harry Feinstone Department of Molecular Microbiology and Immunology, Johns Hopkins Bloomberg School of Public Health, Baltimore, MD, United States

**Keywords:** sex difference, aging, intersectionality, frailty, SARS–CoV–2

## Abstract

Sex differences in the immune system are dynamic throughout the lifespan and contribute to heterogeneity in the risk of infectious diseases and the response to vaccination in older adults. The importance of the intersection between sex and age in immunity to viral respiratory diseases is clearly demonstrated by the increased prevalence and severity of influenza and COVID-19 in older males compared to older females. Despite sex and age biases in the epidemiology and clinical manifestations of disease, these host factors are often ignored in vaccine research. Here, we review sex differences in the immunogenicity, effectiveness, and safety of the influenza and COVID-19 vaccines in older adults and the impact of sex-specific effects of age-related factors, including chronological age, frailty, and the presence of comorbidities. While a female bias in immunity to influenza vaccines has been consistently reported, understanding of sex differences in the response to COVID-19 vaccines in older adults is incomplete due to small sample sizes and failure to disaggregate clinical trial data by both sex and age. For both vaccines, a major gap in the literature is apparent, whereby very few studies investigate sex-specific effects of aging, frailty, or multimorbidity. By providing a roadmap for sex-responsive vaccine research, beyond influenza and COVID-19, we can leverage the heterogeneity in immunity among older adults to provide better protection against vaccine-preventable diseases.

## Introduction

Throughout the lifespan, sex and age are fundamental modifiers of immunity to infectious diseases and the response to vaccination. Females tend to mount stronger immune responses than males (Fish, 2008; Klein and Flanagan, 2016), and immunosenescence leads to impaired immune function and a heightened inflammatory state in older adults (Crooke et al., 2019). There is an important intersection between these host factors, whereby the impact of aging on the immune system differs in males and females (Fink and Klein, 2015; Flanagan et al., 2017). The implications of the interaction between sex and age are clearly demonstrated by the epidemiology and clinical manifestations of respiratory viral diseases, such as influenza and COVID-19 (Giurgea et al., 2021; Scully et al., 2021).

Influenza and COVID-19 represent the largest proportion of the vaccine-preventable diseases that occur in older adults and are thus the focus of this review (Gnanasekaran et al., 2016; Piroth et al., 2021). Despite high coverage with seasonal influenza vaccines in the United States, there are an estimated 4 million incident cases per year in older adults, accounting for 90% of the deaths associated with influenza (Hamborsky et al., 2015; Gnanasekaran et al., 2016). Globally, it has consistently been reported that at older ages, males are at greater risk of infection (Wong et al., 2019), hospitalization (Wang et al., 2002; Crighton et al., 2007; Wang et al., 2015), and mortality (Wang et al., 2002; Azziz-Baumgartner et al., 2013). Similarly, the disproportionate burden of COVID-19 in older adults was recognized early in the pandemic (Kang and Jung, 2020; O’Driscoll et al., 2021), with male sex being a significant predictor of severe outcomes at older ages (Meng et al., 2020; Richardson et al., 2020; Salje et al., 2020; Scully et al., 2020; Bauer et al., 2021).

Vaccines prevent the morbidity and mortality associated with influenza and COVID-19 in older adults. Despite the clear sex and age biases in epidemiology, the impact of these host factors on vaccine responses is often ignored or controlled for in analyses, instead of thoroughly investigated. Here, we review sex differences in the immunogenicity, effectiveness, and safety of influenza and COVID-19 vaccines in older adults, and the available evidence on how sex modifies the impact of age-related factors on vaccine outcomes (Table 1). After identifying major gaps in the literature, we provide a framework for sex-responsive vaccine research to leverage the heterogeneity of older populations to provide optimal protection against vaccine-preventable diseases, beyond influenza and COVID-19.

**TABLE 1:** Summary of sex differences and sex-specific effects of age-related factors on influenza and COVID-19 vaccine outcomes in older adults.

Influenza vaccines	COVID-19 vaccines
Sex differences in older adults	
• Immunogenicity of inactivated influenza vaccines is greater in females than in males	• No sex differences are observed in the immunogenicity of mRNA vaccines
• Evidence of greater VE in females, but insufficient sex- and age-disaggregated data to support a definitive conclusion	• Preliminary evidence that females mount greater antibody responses than males in the context of prior infection
	• No evidence of sex differences in VE
Sex-specific effects of aging	
• Pre-vaccination titers to the high-dose inactivated influenza vaccine decrease with age in males, but not in females	• Both old age and male sex are risk factors for reduced immunogenicity and VE, but sex-specific effects of aging have not been studied
• Antibody titers to the 2009 pandemic H1N1 vaccine decrease with age in females, but not in males	
Sex-specific effects of frailty	
• The impact of frailty on vaccine responses and VE is debated, but no sex-specific effects have been observed	• Both frailty and male sex are associated with reduced VE, but sex-specific effects of frailty have not been studied
Sex-specific effects of comorbidity	
• Not determined	• Not determined

VE as vaccine effectiveness.

### Sex Differences in the Response to Influenza Vaccination in Older Adults

Sex differences in the immune response to influenza vaccination in older adults have been reported for multiple types of inactivated influenza vaccines (IIV). For the standard-dose IIV, older females have greater influenza-specific memory B cells, post-vaccination antibody titers, fold-rises in titers, rates of seroconversion, and rates seropositivity (Cook et al., 2006; Falsey et al., 2009; Voigt et al., 2019; Loeb et al., 2020; Moehling et al., 2020). For the high-dose IIV, which contains four times the amount of antigen as standard dose vaccines and is targeted to older adults, females have greater post-vaccination titers and rates of seroconversion than males (Falsey et al., 2009; Loeb et al., 2020; Moehling et al., 2020). In addition, the 2009 pandemic H1N1 (pH1N1) vaccine generates stronger responses in females in the oldest age groups (Talaat et al., 2010). The consistency of the female-bias in immunogenicity across various vaccine formulations implores continued consideration of sex as a variable of importance in influenza vaccine research.

Sex differences in vaccine effectiveness (VE) in older adults have also been observed, but the evidence is less robust (Gubbels Bupp et al., 2018; Tadount et al., 2019). Looking across seven influenza seasons, VE was significantly greater among females than males, and this difference was more pronounced in older adults (Chambers et al., 2018). A recent systematic review, however, concluded that there is insufficient evidence of a sex difference in effectiveness in older age groups (Tadount et al., 2019). The authors note that many studies are either not designed to assess sex differences or do not present data that is sufficiently disaggregated by age and sex. More evidence is needed to understand how the sex differences in the immunogenicity of influenza vaccines translate to effectiveness.

In terms of vaccine safety, older females consistently report more adverse events (AE) following influenza immunization than males. This has been studied for both the standard-dose (Govaert et al., 1993; Cook et al., 2006; Donalisio et al., 2015) and high-dose (Keitel et al., 2006; Couch et al., 2007) IIV, and has been confirmed in several systematic reviews (Beyer et al., 1996; Cook, 2009; Tadount et al., 2019). In one study that disaggregated data by both sex an age, sex differences were greater at older than younger adult ages (Honkanen et al., 1996). Differences in rates of AE may reflect a gender difference in reporting or a biological sex difference in reactogenicity (Flanagan et al., 2017).

### Sex Differences in the Response to COVID-19 Vaccines in Older Adults

In contrast to the well-documented sex differences in response to influenza vaccination, minimal sex- and age-disaggregated data are currently available to interrogate sex differences in COVID-19 vaccine outcomes in older adults. Published studies including older adults focus primarily on the mRNA vaccines (BNT162b2 and mRNA-1273), and often rely on relatively small sample sizes. In multivariable analysis, female long-term care facility residents (LTCFR) had significantly greater IgG titers and functional antibodies than males after the first mRNA vaccine dose, but not after the second dose (Abe et al., 2021; Brockman et al., 2021). Similarly, in fully vaccinated older adults, there are no significant sex differences in antibody titers (Causa et al., 2021; Kontopoulou et al., 2021; Ríos et al., 2021). Among LTCFR who recovered from SARS-CoV-2 infection, however, there is a trend of higher antibody levels in females than males (Canaday et al., 2021). While sex differences in immune responses are currently not apparent among older adults who received mRNA vaccines, data are missing for other vaccine platforms, and more research is needed to understand how sex differences may be modified by prior infection or affect immunity against variants of concern (e.g., Omicron).

The COVID-19 vaccine clinical trials revealed remarkably high efficacy against the ancestral virus at all ages but did not provide estimates disaggregated by sex within each age group (Baden et al., 2020; Polack et al., 2020; Falsey et al., 2021; Sadoff et al., 2021). Sex differences observed in COVID-19 outcomes in unvaccinated older adults are not observed in fully vaccinated populations, such that VE with respect to hospitalization and mortality is the same in males and females (Gomes et al., 2021). Similarly, sex does not impact the risk of breakthrough infection in LTCFR (Hollinghurst et al., 2021) or in the general older adult population (Antonelli et al., 2021; Havers et al., 2021). Like immunogenicity, sex differences in COVID-19 VE are currently not apparent in older adults, but failure to disaggregate data by both sex and age may be obscuring an important effect.

Few studies have provided both sex- and age-disaggregated safety data for the SARS-CoV-2 vaccines. Among older adults, local, systemic, and medically attended AE are more common in females than in males (Choi et al., 2021; Hoffmann et al., 2021). Both among females and overall, older individuals report fewer AE than younger individuals (Choi et al., 2021; Hoffmann et al., 2021), but the proportion of events classified as serious increases with age (Xiong et al., 2021). Sex differences have been reported for several serious AE, including a female bias in anaphylaxis (Blumenthal et al., 2021; Shimabukuro et al., 2021; Somiya et al., 2021) and thrombosis with thrombocytopenia syndrome (Lai et al., 2021), and a male-bias in myocarditis (Klein et al., 2021), but these events predominantly occur in younger age groups. It is currently unclear what age- and sex-dependent protective factors may explain the absence of these events following immunization in older populations.

### The Intersection of Sex With Age-Related Factors

In addition to sex differences in vaccine outcomes in older adults, sex can also modify the effect of chronological age, frailty, or the presence of comorbidities on immunogenicity or VE. Intersectional analyses investigating differences among males and females caused by age-related factors are crucial to a robust understanding of the heterogeneity of vaccine responses.

### The Intersection of Sex and Aging

Age-associated changes in immunity (e.g., heightened pro-inflammatory state and deficits in both cellular and humoral immunity) (Pinti et al., 2016; Bulati et al., 2017; Crooke et al., 2019) are coupled with changes in the hormonal milieu in both males and females, which can cause sex-specific effects of aging (Gubbels Bupp et al., 2018). For example, decreasing concentrations of estrogens with menopause are associated with reduced B and T cell numbers and lower concentrations of IL-6 in females (Giglio et al., 1994; Kamada et al., 2001; Kumru et al., 2004), while decreases in testosterone in males are inversely correlated with levels of soluble IL-6 receptor (Maggio et al., 2006). Furthermore, profiling of peripheral blood mononuclear cells across the lifespan revealed that the decline in naïve T cell activity and increase in monocyte function observed with age occur to a greater extent in males than females, and are accompanied by a male-specific decline in B cell transcriptional activity (Márquez et al., 2020). This analysis also found that abrupt age-associated epigenetic changes occur earlier in males than females, and that while older females have higher adaptive immune cell activity, older males have higher inflammatory and monocyte activity (Márquez et al., 2020). These findings have been replicated in multiple other studies (Wikby et al., 2008; Goetzl et al., 2010; Hirokawa et al., 2013; Marttila et al., 2013; Furman et al., 2014), and together suggest that the effects of aging on the immune system are dampened and occur at a slower rate in females than males.

Sex-specific effects of aging have been reported in the humoral immune response to influenza vaccination. In the case of repeated vaccination with the high-dose IIV, pre-vaccination titers to A/H3N2 and influenza B viruses decrease with age in males but not in females, suggesting that older females enter each influenza season with greater immunity than their male counterparts (Shapiro et al., 2021b). In contrast, in response to the pH1N1 vaccine, age-associated declines in immunogenicity are not observed in males, but are observed in females, where they are associated with declining concentrations of estradiol (Potluri et al., 2019). Although the results of these studies may appear conflicting, it is important to note that the high-dose IIV study included only older adults (aged ≥75 years), while the pH1N1 study compared younger (aged 18–45 years) and older (aged ≥65 years) cohorts. Furthermore, pandemic viruses and vaccines provide a unique opportunity to evaluate responses to a novel viral antigen, whereas during seasonal influenza vaccination, the influence of prior exposure to influenza on vaccine immunogenicity may be sex differential (Shapiro et al., 2021b). Thus, the discordance in the two studies may be explained by the pandemic versus seasonal nature of the vaccines investigated.

For COVID-19 vaccines, multiple studies that either control for or ignore sex have reported that vaccine-induced antibody responses decrease with age in older adults (Abe et al., 2021; Bates et al., 2021; Canaday et al., 2021; Causa et al., 2021; Collier et al., 2021; Jabal et al., 2021). There is conflicting evidence on the effect of age on VE, with some studies reporting a negative effect (Bajema et al., 2021; Robles-Fontan et al., 2021; Cerqueira-Silva et al., 2022), and others reporting no effect (Aldridge et al., 2021; Chemaitelly et al., 2021; Haas et al., 2021). For antibody responses (Levin et al., 2021; Lustig et al., 2021) and VE (Agrawal et al., 2021; Liu et al., 2021; Nordström et al., 2021), several studies have identified both male sex and old age as risk factors, but have not investigated sex-specific effects of aging, with the result that it is currently unknown whether the effects of aging on COVID-19 vaccine outcomes differ in males and females.

### The Intersection of Sex and Frailty

Frailty is defined as reduced physiological function leading to increased vulnerability and is associated with profound immune dysregulation that can impact vaccine responses in a sex-specific manner (Chen et al., 2014; Chen et al., 2019; Gordon and Hubbard, 2020). Importantly, the prevalence of frailty is higher, but mortality is lower, in older females than older males, suggesting fundamental sex differences in pathophysiology (Gordon et al., 2016; Park and Ko, 2021). For example, frailty is associated with increased frequency of pro-inflammatory late memory B cells only in males (Nevalainen et al., 2019), and baseline concentrations of CRP and fibrinogen are associated with increased incidence of frailty only in females (Gale et al., 2013). The relationship between frailty and vaccine responses is debated in the literature, and few studies have considered how sex may modify this relationship.

For influenza, frailty is not associated with pre- or post-vaccination HAI titers in either males or females, nor is a sex difference in the impact of frailty observed (Moehling et al., 2018; Shapiro et al., 2021b). In addition, no association between frailty and antibody responses is observed either when controlling for sex in statistical analysis (Narang et al., 2018; Moehling et al., 2020) or when ignoring sex altogether (DiazGranados et al., 2015; Bauer et al., 2017; Epps et al., 2017). In contrast, it has also been reported that frailty has both a negative (Yao et al., 2011) and a positive effect on antibody responses (Loeb et al., 2020), and that frailty may impact measures of vaccine-induced cell-mediated immunity (Moehling et al., 2020). Evidence of the effect of frailty on influenza VE is also conflicting, with one study reporting that VE decreases significantly with frailty (Andrew et al., 2017), and another reporting that VE estimates are not confounded by frailty (Talbot et al., 2016).

The impact of frailty on COVID-19 vaccine outcomes has only been investigated without consideration of sex. Frailty does not impact vaccine-induced antibody responses against BNT162b2 when controlling for sex and other covariates (Ríos et al., 2021) or when ignoring sex (Demaret et al., 2021; Seiffert et al., 2021). Frailty does, however, increase the risk of post-vaccination infection when controlling for sex and age (Antonelli et al., 2021; Hollinghurst et al., 2021). In analyses that control for sex, living in a long-term care facility, a proxy for frailty, was associated with increased risk of severe COVID-19 outcomes post-vaccination (Agrawal et al., 2021) and individuals with breakthrough infections were more likely to be LTCFR than unvaccinated infected individuals (Havers et al., 2021). Finally, VE is lower and wanes faster in both frail individuals and males, but sex-specific effects of frailty were not investigated (Nordström et al., 2021). Together, the data support a role for frailty in impairing COVID-19 VE beyond the impact of chronological age, but whether this effect is different in males or females remains unknown.

For both influenza and COVID-19, the frailty literature is complicated by different methods used to measure frailty, small sample sizes, and lack of consideration of biological sex. More research is needed to address the discordance and gaps in the literature.

### The Intersection of Sex and Comorbidity

There is a high prevalence of comorbid conditions in older adults, which can have immunomodulatory effects that impact infectious disease epidemiology and vaccine responses (Kwetkat and Heppner, 2020). For example, the prevalence of health conditions that increase the risk of influenza-related complications (e.g., chronic pulmonary or cardiovascular disease, metabolic disorders, etc.) rises drastically with age and is significantly higher in older males than older females (Zimmerman et al., 2010). Similarly, a greater percentage of older males than females are at high-risk of requiring hospitalization if infected with COVID-19 due to the presence of an underlying condition (e.g., cardiovascular disease, diabetes, cancer, etc.) (Clark et al., 2020).

Despite the clear age-by-sex bias in the prevalence of comorbidities that may impact influenza and COVID-19 vaccine responses, sex-specific effects of chronic conditions have not been studied. For influenza vaccines, studies that either control for or do not consider sex report that influenza vaccine immunogenicity (Falsey et al., 2009) and relative VE (DiazGranados et al., 2015; Boikos et al., 2021) do not differ by the presence of high-risk conditions. For COVID-19 vaccines, analyses that control for sex reveal that multimorbidity is not associated with immunogenicity in older adults (Causa et al., 2021; Ríos et al., 2021), but is associated with VE (Agrawal et al., 2021; Butt et al., 2021; Havers et al., 2021; Lewis et al., 2021; Nordström et al., 2021). These data reveal a gap in the literature, whereby the role of sex in modifying the effect of multimorbidity on vaccine responses remains poorly understood.

## Discussion

**FIGURE 1:**
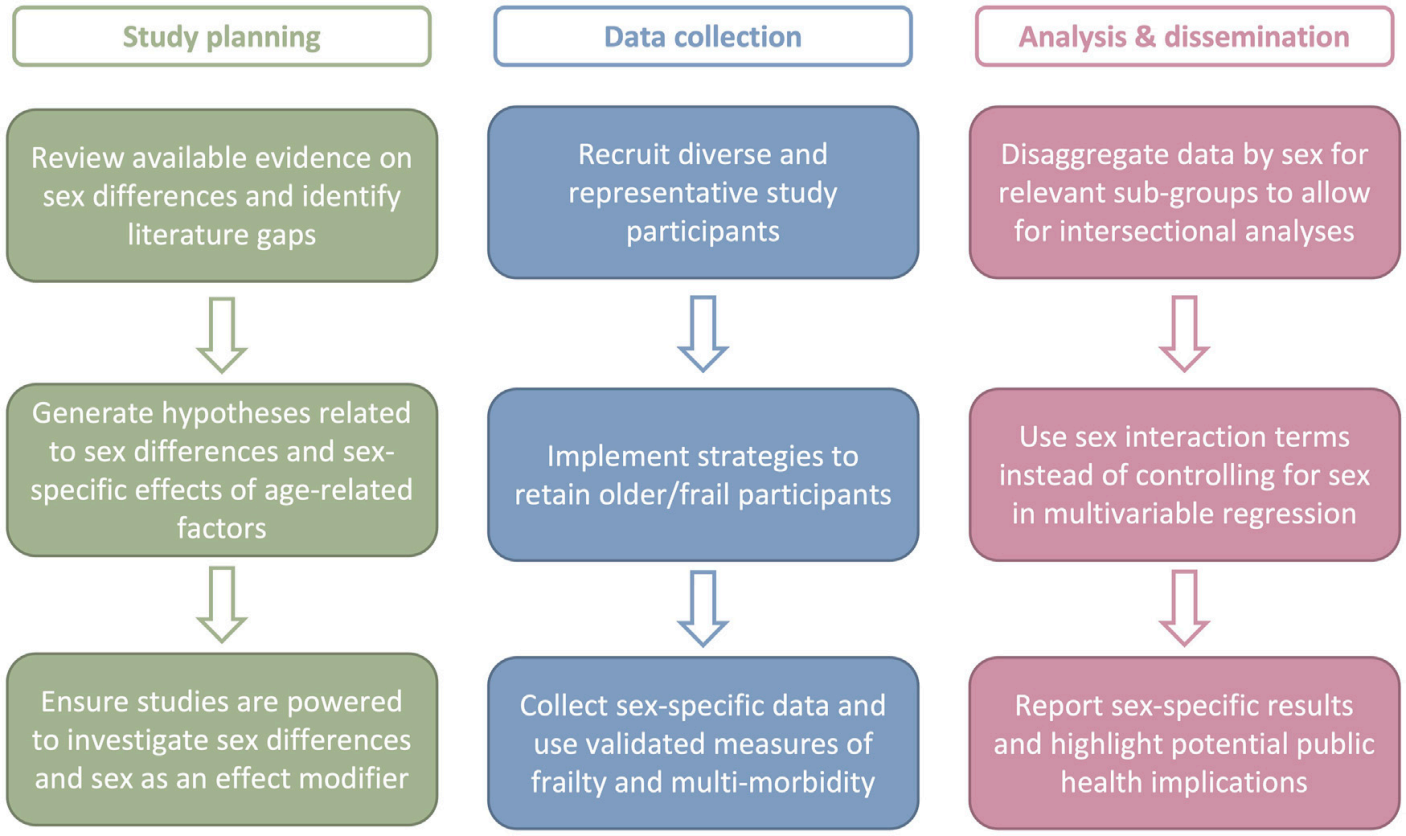
Roadmap for sex-responsive vaccinology research in older adults. Sex-responsive vaccine research in older adults requires careful thought and at the study planning, data collection, analysis, and dissemination phases. Action items for each phase are provided.

Both sex and age-related factors have important consequences on vaccine responses in older adults, but the intersection of sex with age, frailty, and comorbidity remain incompletely elucidated (Table 1). This literature gap suggests that a roadmap is needed for sex-responsive vaccinology research in older adults (Figure 1). Sex-responsive research requires careful thought at the study planning, data collection, analysis, and dissemination phases.

First, study planning must begin with a review of the literature to identify gaps and generate hypotheses related to sex differences and sex-specific effects. Following hypothesis generation, sample size calculations are required to adequately power studies for sub-group analyses and interrogation of sex as an effect-modifier (Shapiro et al., 2021a). In the literature reviewed above, small sample sizes suggest that many studies are not appropriately powered, and are thus prone to type II errors, whereby the null hypothesis of no sex difference is erroneously accepted (Columb and Atkinson, 2016). Larger sample sizes, with adequate numbers of males and females, are thus necessary for correct statistical inference. Instead of interpreting larger sample sizes as a burden, sex should be viewed as an important modifier of vaccine-induced immunity and outcomes that could improve study design and interpretation (Klein et al., 2015).

Second, recruitment of diverse participants and inclusion of populations that are typically under-represented in research (e.g., populations of color, frail individuals, gender minorities) is essential. Once recruited, explicit strategies are needed for participant retention. For example, home visits that do not require participants to travel to study sites are an effective method to retain participants with reduced mobility. Data collection should also utilize validated measures of frailty, and multi-morbidity, along with sex-specific questions (e.g., use of hormone replacement therapy) to thoroughly understand underlying differences among and between male and female participants.

Third, sex must be considered as a variable of importance, rather than a confounder to be controlled for, during data analysis and dissemination of results (Shapiro et al., 2021a). This begins by disaggregating data by sex for relevant sub-groups (i.e., age, frailty status). In formal analysis, use of interaction terms between sex and other variables allows for interrogation of how trends differ between males and females. Finally, dissemination of results should underscore whether findings are true for both males and females and highlight the clinical and public health implications of any sex-specific findings.

In conclusion, we identified sex differences in influenza and COVID-19 vaccine outcomes in older adults but uncovered a significant gap in the literature in terms of the sex-specific effects of age-related factors. While the present review focused on influenza and COVID-19 vaccines, the conclusions and research roadmap extend to other vaccines administered to older adults, such as the herpes zoster and pneumococcal vaccines, as well as other public health interventions. Implementation of the roadmap requires engagement at all levels, including funders, regulatory agencies, vaccine manufacturers, and academic institutions. Ultimately, it is through sex-responsive research that we can leverage the heterogeneity of older populations to provide optimal protection against vaccine-preventable diseases.
